# Simulation of Slip the Velocity Effect in an AC Electrothermal Micropump

**DOI:** 10.3390/mi11090825

**Published:** 2020-08-31

**Authors:** Fraj Echouchene, Thamraa Al-shahrani, Hafedh Belmabrouk

**Affiliations:** 1 Electronic and Microelectronics Laboratory, Department of Physics, Faculty of Science of Monastir, University of Monastir, Monastir 5000, Tunisia; frchouchene@yahoo.fr; 2 Department of Physics, College of Science, Princess Nourah Bint Abdulrahman University, Riyadh 11671, Saudi Arabia; thmalshahrani@pnu.edu.sa; 3 Department of Physics, College of Science at Zulfi, Majmaah University, Majmaah 11952, Saudi Arabia

**Keywords:** slip velocity, AC electrothermal, micropump, microfluidics

## Abstract

The principal aim of this study was to analyze the effect of slip velocity at the microchannel wall on an alternating current electrothermal (ACET) flow micropump fitted with several pairs of electrodes. Using the finite element method (FEM), the coupled momentum, energy, and Poisson equations with and without slip boundary conditions have been solved to compute the velocity, temperature, and electrical field in the microchannel. The effects of the frequency and the voltage, and the electrical and thermal conductivities, respectively, of the electrolyte solution and the substrate material, have been minutely analyzed in the presence and absence of slip velocity. The slip velocity was simulated along the microchannel walls at different values of slip length. The results revealed that the slip velocity at the wall channel has a significant impact on the flow field. The existence of slip velocity at the wall increases the shear stress and therefore enhances the pumping efficiency. It was observed that higher average pumping velocity was achieved for larger slip length. When a glass substrate was used, the effect of the presence of the slip velocity was more manifest. This study shows also that the effect of slip velocity on the flow field is very important and must be taken into consideration in an ACET micropump.

## 1. Introduction

Alternating current electrokinetics (ACE) such as AC electrothermal (ACET), AC electro-osmosis (ACEO), and dielectrophoresis (DEP) are a promising technology for development in lab-on-a-chip systems and Bio-MEMS [1,2,3,4,5,6,7]. Dielectrophoresis (DEP) has been extensively studied in order to manipulate particles, capture DNA, and perform cell separation [3]. AC electro-osmosis (ACEO) has been used for driving, mixing, or pumping in several kinds of biochips or micro-fluidic chips under low frequencies [2,7]. AC electrothermal flow has been tremendously well-used for biological applications in low voltages and at higher frequencies, i.e., improvement in quality and performance of heterogeneous immuno-sensors in microfluidic systems [5], DNA hybridization [1], and on-chip mixing of fluids [8]. In previous studies, we used the ACET flow combined with obstacles inside the microchannel in order to improve the binding efficiency of an immunoassay for a biosensor [9,10]. The effect of the slip velocity was ignored. Selmi and Belmabrouk [11] investigated the repercussions of the AC electroosmosis force on a microfluidic biosensor. The main purpose was to prove how the AC electroosmosis could modify the flow topology and improve the binding reaction inherent to the biosensor.

Pumping of electrolytes using the ACET has important relevance for microfluidic devices [12,13,14,15]. The pumping using the AC electrokinetics is characterized by low power consumption and the electrodes can be inserted inside the microfluidic devices. ACET electrothermal flow is commanded by an electrostatic force created by the local gradient of the temperature and the electrical conductivity (σ) and permittivity (ε) of the fluid, while taking into account the interaction induced by the electric field [16]. The gradients of the physical parameters of the fluid (such as σ, ε, dynamic viscosity μ, and density ρ) are actually caused by the non-uniform distribution of the temperature which is itself due to Joule heating [17].

In recent years, ACET flow has garnered increasing interest worldwide regarding fundamental and applied aspects. ACET flow was initiated by Green et al. [18] in the wake of the experimental investigation of ACET flow in microfluidic chips containing microelectrode pairs. Hong et al. [19] proposed a two-dimensional study of ACET flow. The microchannel contains asymmetrical planar electrode pairs. This configuration is similar to an ACEO pump. In a complementary study, Hong et al. [20] realized a numerical study of an ACET micropump. The thermal, physical, and electrical parameters of the fluids are considered variable versus the temperature. Two ACET pump designs with biobuffers such as lysogeny broth medium at 0.754 S/m and minimal salts medium at 0.145 S/m were optimized by Wu et al. [21]. High-efficiency pumping was achieved for T array. An improvement of AC electrothermal micropumping was studied by Du and Manoochehri [22,23]. The capacity of micro-grooved surface channels has been proved at low voltage. Zhang et al. [24] investigated a two-phase ACET micropump fitted with a coplanar asymmetric electrode array. Using this device, the fluid flow rates can be 50% faster than those obtained with a single-phase structure.

Gao and Li [25] studied an ACET micropump integrating an asymmetric spiral microelectrode pair in a cylindrical microchannel in order to drive rapidly and to mix efficiently, high-conductivity fluids. Salari et al. [26] proposed an original AC multiple-array electrothermal micropump. In a recent study, Salari and Dalton [27] introduced a double-array ACET equipment involving two opposite microelectrode arrangements. This device can be used for simultaneous pumping and mixing. Ren [28] has investigated the microfluidic pumping of blood flow. The blood is considered a non-Newtonian fluid. The ACET force is taken into account. The immersed boundary-lattice Boltzmann method (IB-LBM) and the hybrid boundary element method (BEM) have been used.

The slip velocity phenomenon at the liquid/solid interface, observed in polymer solutions [29,30], colloidal particles [31], and considerably in red blood cell suspensions [31], plays an important role in electro-osmotic flow through micro-channels.

Goswami and Chakraborty [32] theoretically studied the electro-osmotic flow in a micro-channel using a slip velocity condition. Zhao and Yang [33] have investigated the combined effects of streaming potential and hydrodynamic Navier slip on pressure-driven flows in a micro-channel. Rivero and Cuevas [34] analyzed fluid/wall slippage in magnetohydrodynamic (MHD) micropumps for low-Hartmann-number flow. The influence of the slip length on the flow rate was tested. Shit et al. [35] have studied the effects of slip velocity on rotating electro-osmotic flow in a non-uniform micro-channel.

It has been observed that the no-slip condition is not valid whatever the configuration—in particular, in many microfluidic and nanofluidic applications whose hydraulic diameter ranges from 10 μm to 200 μm [36]. The interfacial slip is characterized by a Navier-slip condition and the slip velocity is written against the normal gradient of the tangential velocity component and the tangential temperature gradient at the interface.

In the current paper, two-dimensional models available in the literature for describing the AC electrothermal flow in micropump are revisited. The numerical study considers slip and no-slip boundary conditions. These models have been used to investigate an ACET flow micropump having asymmetric planar electrode pairs and using slip and no-slip boundary conditions. The effects of frequency, voltage, concentration, and substrate material under slip velocity at the wall of the microchannel were also discussed.

## 2. Physical Configuration

**Figure 1:**
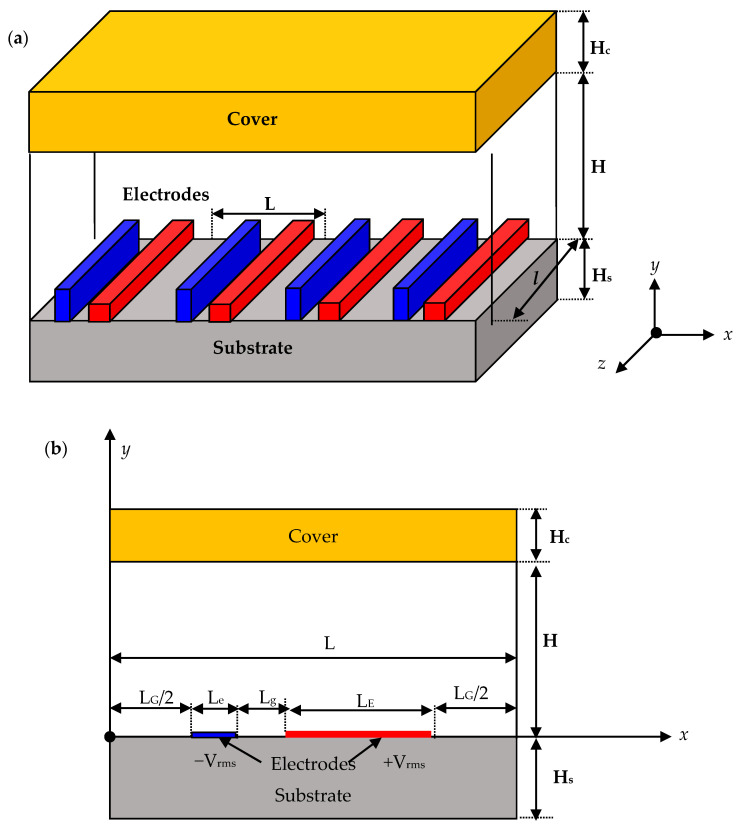
Geometry of an alternating current electrothermal (ACET) micropump involving the electrodes. (**a**) 3D view, (**b**) 2D view of the reduced computational domain.

Figure 1a displays the three-dimensional configuration of an ACEO micropump having asymmetric pairs of interdigitated electrodes fabricated on the substrate. The consecutive electrodes are supposed to be in antiphase. Therefore, there is no need to use complex notation and only the real parts of AC quantities are computed. We suppose that the channel height H is negligible compared to the length *l* of the electrode. Considering the periodicity of the asymmetric electrodes, we can, therefore, reduce the 3-D geometry to a 2D geometry (x, y) [19,20]. Only one pair of electrodes is involved, as indicated in Figure 1b.

The dimensions of the computational domain of the micropump were taken from the literature: a microchannel height H = 50 µm and length L = 60 µm. The substrate (glass or silica) is of thickness H_s_ = 500 µm and the cover (PDMS) is of thickness H_c_ = 100 µm. A pair of electrodes of small thickness is on the bottom of the microchannel. L_e_ and L_g_ are the smaller electrode’s width and gap, respectively. L_E_ and L_G_ are the larger electrode’s width and gap. An AC electric field is applied through the electrodes. The nanofluid used is the KCl electrolyte solution. The physical properties of KCl, the cover, and the substrate are summarized in Table 1.

## 3. Physical Model

In order to analyze the electrothermal mechanism, the mathematical formulation requires solving the Navier–Stokes, heat, and Poisson equations in order to find the velocity, the temperature, and the electrical field. As mentioned above (Figure 1b), a 2D configuration is considered in this work.

The Poisson equation that governs the electrical potential is solved in Cartesian coordinates:

(1) $$\frac{\partial^{2}\varphi }{\partial x^{2}}+\frac{\partial^{2}\varphi }{\partial y^{2}}=0$$

where φ indicates the root mean square (rsm) voltage, and *x*, *y* denotes the Cartesian coordinates. Since the relative permittivity of the fluid is considered to be independent of the temperature, the electrical field E is easily obtained from the electrical potential. The boundary conditions related to the above equation are:

- At the left and right walls (x=0 and x=L), a periodic condition is applied.
- At the anode and the cathode, the potential is respectively equal to $-V_{\mathrm{rsm}}$ and $V_{\mathrm{rms}}$ where $V_{\mathrm{rms}}$ is an adjustable value.
- At the other walls (y=H and the remaining part of y=0), an insulation condition is applied.

On the other hand, by neglecting the heat convection, the energy equation in all domains can be expressed as:

(2) $$\{\begin{array}{l} \lambda \left(\frac{\partial^{2}T}{\partial x^{2}}+\frac{\partial^{2}T}{\partial y^{2}}\right)+\langle \sigma E^{2}\rangle \;=0\;\;\;\;in\;the\;fluid\;region\\ \frac{\partial^{2}T}{\partial x^{2}}+\frac{\partial^{2}T}{\partial y^{2}}=0\;\;\;\;\;in\;the\;cover\;and\;the\;substrate \end{array}$$

where σ and λ are respectively the electrical and thermal conductivities of the fluid. Those quantities are assumed to be constant. The term σ × E^2^ is the volumetric source term due to Joule heating, and the symbol 〈〉 represents the time-averaged value. The boundary conditions related to the heat equation are:

- At the left and right walls (x = 0 and x = L), the inlet and the outlet walls are assumed to be adiabatic. The heat flux is equal to zero at these boundaries.
- At the walls (y = 0 and y = H), the temperature is assumed to be continuous.
- At the external walls of the cover and the substrate ($y\;=\;-H_{s}$ and $y\;=\;H\;+\;H_{c}$), an isothermal condition is applied: $T_{s}=T_{c}=298\;K$.

With regard to the flow velocity field in the microfluidic channel, it is governed by the continuity and Navier–Stokes equations. The continuity equation is given by:

(3) $$\frac{\partial u}{\partial x}+\frac{\partial v}{\partial y}=0$$

To write the Navier–Stokes equations involving the electrothermal force, we assume that the fluid is incompressible, that the flow is steady, and that the flow occurs at low Reynolds numbers. It is straightforward to obtain:

(4) $$\{\begin{array}{c} -\frac{\partial p}{\partial x}+\mu \left(\frac{\partial^{2}u}{\partial x^{2}}+\frac{\partial^{2}u}{\partial y^{2}}\right)+<F_{ex}>=0\\ -\frac{\partial p}{\partial y}+\mu \left(\frac{\partial^{2}v}{\partial x^{2}}+\frac{\partial^{2}v}{\partial y^{2}}\right)+<\;F_{ey}\;>=0 \end{array}$$

Here, *p* is the pressure, and *u* and *v* are the velocity components. The fluid is Newtonian and its viscosity is *μ* = 10^−3^ Pa·s. The temperature rise is less than 2 K. Therefore, the viscosity may be considered as constant during the simulations. No effects of heat convection or buoyancy take place.

The two electrodes produce an AC electric field which creates the electrothermal force per unit volume $\langle \vec{F_{e}}\rangle$ [9,10,37]. Its expression is derived using a linear perturbation method:

(5) $$\langle \vec{F_{e}}\rangle =\frac{1}{2}\varepsilon \left[\frac{\left(\alpha -\beta \right)}{1+{\left(\omega \tau \right)}^{2}}\left(\vec{grad}T.\vec{E}\right).\vec{E}-\frac{1}{2}\alpha {\left|E\right|}^{2}\vec{grad}T\right]$$

α and β represent the expansion coefficients of the fluid permittivity ε and the electrical conductivity σ, respectively. They are given by:

$$\alpha =\frac{1}{\varepsilon }\frac{\partial \varepsilon }{\partial T}\;and\;\beta =\frac{1}{\sigma }\frac{\partial \sigma }{\partial T}$$

For water, the values of these parameters are *α* = −0.004 K^−1^ and *β* = 0.02 K^−1^ [19]. *ω* represents the electric field angular frequency. τ = ε/σ is the relaxation time of the liquid.

The boundary conditions related to the Navier–Stokes equations are:

- At the left and right walls (x = 0 and x = L), the normal stress is equal to zero at the inlet and outlet walls.
- At the walls (y = 0 and y = H), a slip or no-slip velocity condition is applied.

It should be mentioned that the Poisson and Navier–Stokes equations are solved only inside the microchannel, while the heat equation is solved throughout the structure.

In order to analyze the effect of slip velocity on 2D ACE electrothermal flow, we use the following expressions [38]:

(6) $$u_{slip}=u-u_{w}=\Lambda \frac{\left(2-\alpha_{v}\right)}{\alpha_{v}}{\left(\frac{\partial u}{\partial y}+\frac{\partial v}{\partial x}\right)|}_{w}+{\frac{\sigma_{T}\mu }{\rho T}\frac{\partial T}{\partial x}|}_{w}$$

The first term in Equation (6) is due to the velocity gradient normal to the surface, whereas the second term results from the temperature gradient along the surface. In this study, the tangential temperature gradient is very low; the second term can be ignored. *u_w_* is the solid wall velocity assumed to be equal to zero, Λ is the molecular mean free path, *α_v_* is the tangential momentum accommodation coefficient (TMAC) varying from 0.0 to 1.0, and *σ_T_* = 0.75 [39] is the thermal slip coefficient. L_s_ = Λ (2 − α*_v_*)/α*_v_* is the slip length which experiments have shown that can take values ranging from nanometers to microns [40,41].

The computations were carried out using the finite element method. Applying the Galerkin weighted residual method, Equations (2)–(4) can be transformed into a matrix form as

(7) KX=Q

where *X* is the unknown vector; K and Q are the stiffness matrix and load vector, respectively.

**Figure 2:**
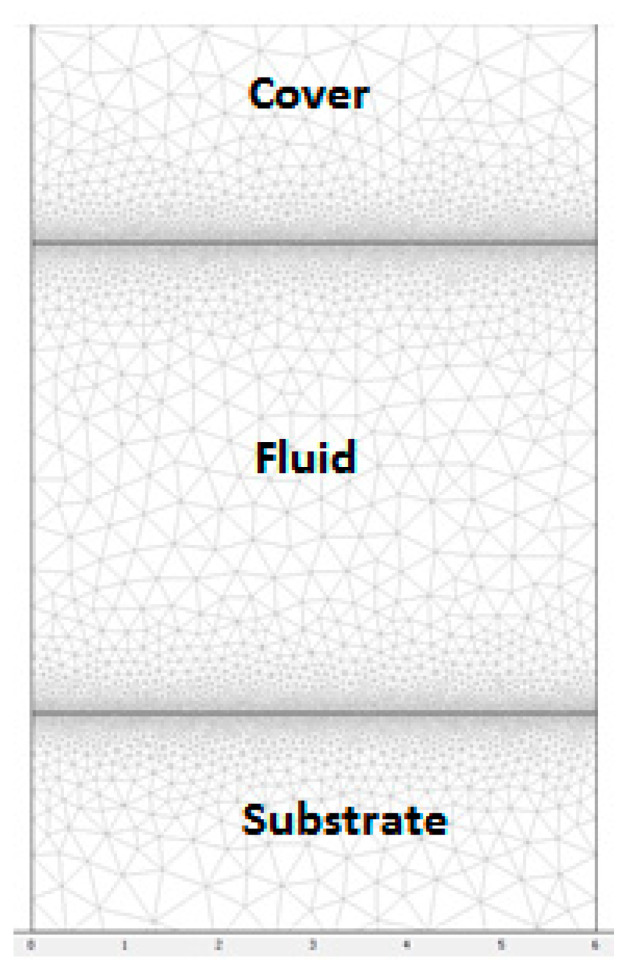
2D unstructured mesh with triangular elements; the mesh is refined near the wall of microchannel.

First, the electric potential is solved using Equation (1). Then the resulting thermal field is calculated from Equation (2), and finally the induced flow velocity is solved from the Navier–Stokes equation, Equations (3) and (4). A triangular mesh containing 15,988 triangular elements was used for all our simulations. The mesh refinement was especially achieved close to the electrodes. Figure 2 shows this mesh where the region near the wall of the microchannel is refined for a better resolution.

## 4. Results and Discussion

### 4.1. Effects of Slip Velocity on Characteristics of Electrothermal Flow

**Figure 3:**
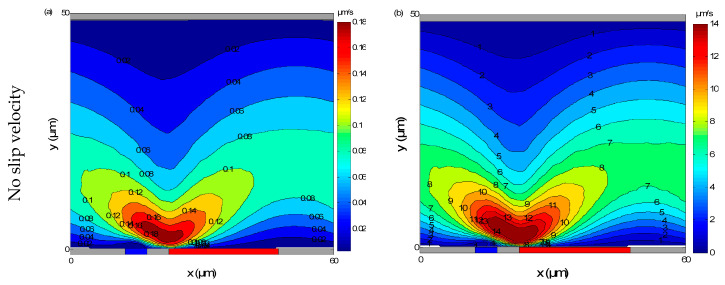

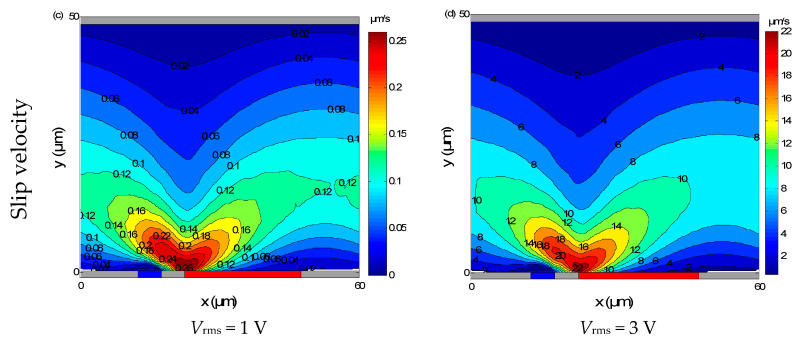
Velocity contours, without slip velocity for *V*_rms_ = 1 V (**a**) and *V*_rms_ = 3 V (**b**) and with slip velocity for *V*_rms_ = 1 V (**c**) and *V*_rms_ = 3 V (**d**). The slip length and the frequency are taken as equal to *L_s_* = 1µm and *f* = 100 kHz, respectively. The slip velocity has an effect on the distribution of fluid velocity. An extensive increase in the velocity field due to the increase of the shear stress is noted.

Numerical results for the velocity fields taking into account the electrothermal force and with or without slip velocity are presented in Figure 3; computations were performed for a microchannel containing a glass substrate with *L*_e_ = *L*_g_ = 5 μm and *L*_E_ = *L*_G_ = 25 μm. The electrical conductivity σ was equal to 0.01 S/m and the frequency *f* was 100 kHz, whereas the RMS voltage *V*_rms_ was 1 or 3 V. The slip velocity boundary condition was applied on the top and bottom walls of the microchannel for a slip length *L*_s_ = 1 µm. In Figure 3, the smaller and larger electrodes are indicated in blue and red, respectively. The electrothermal flow was generated at the narrow electrode. Then it was oriented to the wide electrodes. Furthermore, the velocity reached its maximal value at the electrode borders and the velocity decayed away from the bottom wall because of the viscous effects. The influence of the slip velocity on the fluid flow is presented on Figure 3c,d for *V*_rms_ = 1 V and 3 V, respectively. It is clear that the slip velocity has an effect on the distribution of fluid velocity. We note an extensive increase in the velocity field due to the increase of the shear stress.

From Figure 3c,d, it appears that the effects of slip velocity on the flow field depend mainly on the applied voltage. The increase of the velocity field is more and more important when *V*_rms_ is large.

**Figure 4:**
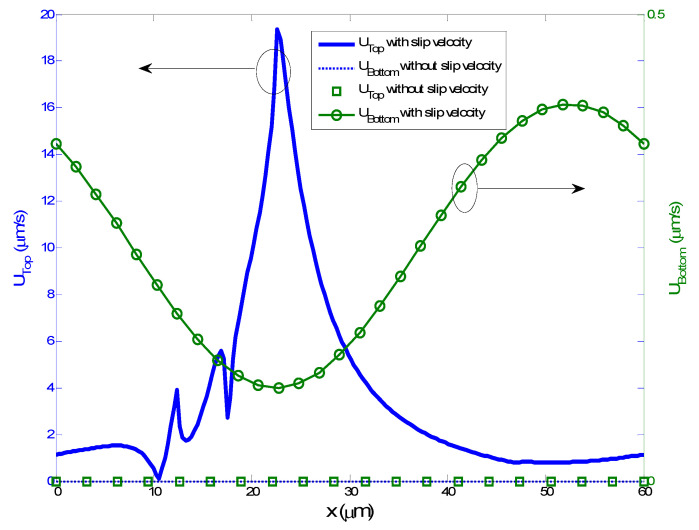
Slip velocity profile on the top and bottom walls for *V*_rms_ = 3 V and *L*_s_ = 1 µm compared to the no-slip boundary condition.

In order to evaluate the impact of the slip condition at the wall on the velocity field, we plotted the slip velocity at both upper and down boundary walls of the microchannel with and without the slip condition for *V*_rms_ = 3 V and *L*_s_ = 1 µm (Figure 4). The slip velocity under the no-slip condition was equal to zero because the wall shear stress was set to zero. Figure 4 also shows that the biggest slip velocity took place at the bottom wall and more precisely near the small region between the electrodes, where the shear stress is important. In addition, the slip velocity at the top wall is also very small due to the viscous effects.

**Figure 5:**
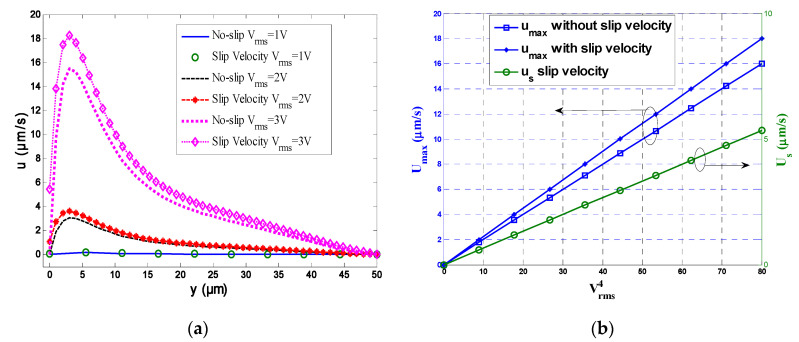
(**a**) Transversal velocity profile at x = 20 μm for *V*_rms_ = 1, 2, and 3 V; (**b**) maximum velocity and slip velocity profiles as a function of $V_{\mathrm{rms}}^{4}$ with and without slip velocity.

Figure 5a shows the transversal velocity distribution at x = 20 µm for *V*_rms_ = 1, 2, or 3 V and in the presence or not of a slip velocity. The slip length was fixed to 0.5 µm. It appears that the maximum velocities occurred at y ≈ 3 μm. This was valid for the three voltages and for both kinematic conditions (with or without slip). With the increase in voltage, the slip velocity and the maximum velocity increase rapidly. From this figure, we can conclude that the maximum velocity for slip and no-slip and slip conditions is proportional to $V_{\mathrm{rms}}^{4}$ (Figure 5b) which is in accordance with the existing results in the literature [19,20]. The discrepancy in maximum velocity between the no-slip and slip conditions increases when *V*_rms_ increases.

**Figure 6:**
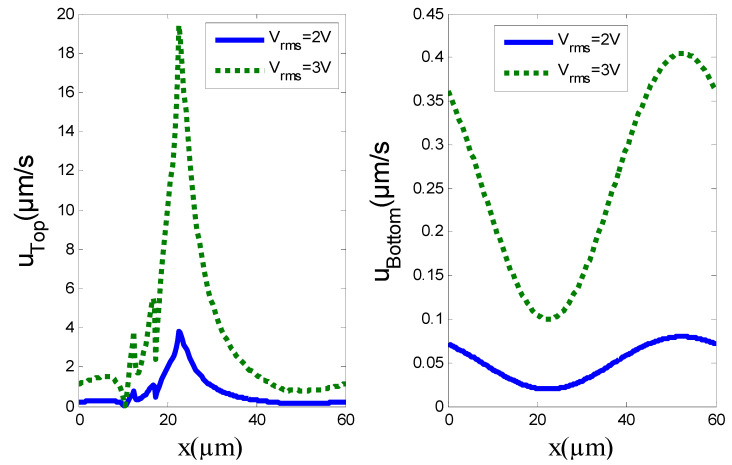
Slip velocity profile on the top and bottom walls for *V*_rms_ = 2 or 3 V and *L*_s_ = 1 µm.

In order to grasp the impact of the applied voltage *V*_rms_ on the slip velocity at the wall, the slip velocities on the top and bottom walls have been plotted for *V*_rms_ = 2 and 3 V, respectively (Figure 6). The profiles are somewhat similar. However, when the voltage increases, the maximum slip velocity exhibits a sharp increase. The same profiles were observed with a significant increase in the maximum slip velocity when the applied voltage increased.

### 4.2. Slip Velocity’s Effect on the Average Pumping Velocity

The average pumping velocity u_ave_ can be defined according to:

(8) $$u_{\mathrm{ave}}=\frac{1}{H}\int_{0}^{H}udy$$

**Figure 7:**
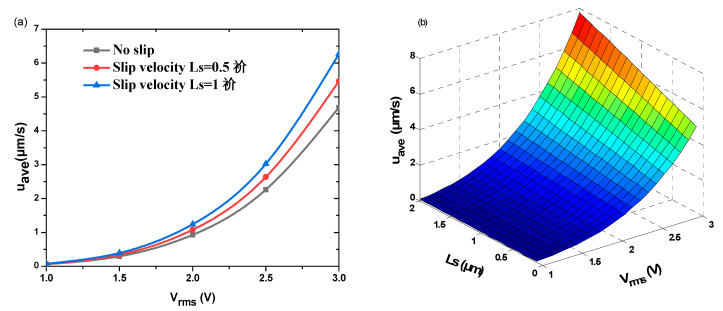
(**a**) Average pumping velocity versus *V*_rms_, with and without slip velocity, for *L*_s_ = 0.5 µm and 1 µm. (**b**) Isovalues of u_ave_ as a function of *L*_s_ and *V*_rms_.

It is worthwhile to discuss how the slip velocity and the applied voltage can affect the average velocity u_ave_. Figure 7 shows the effects of these two parameters on the average velocity u_ave_. It can also be seen from Figure 7b that the u_ave_ increases almost linearly with the slip length. The maximum average velocity was achieved for larger *L*_s_ and larger *V*_rms_.

### 4.3. Effects of Slip Velocity and Electrical Conductivity on Average Velocity

**Figure 8:**
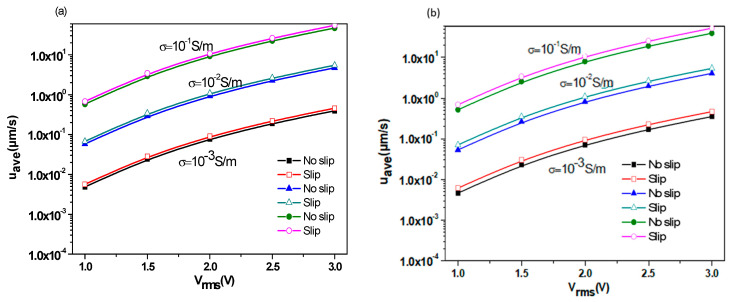
The effect of electrolyte conductivity on average velocity without slip velocity and with slip velocity for (**a**) *L*_s_ = 0.5 µm and (**b**) *L*_s_ = 1 µm.

Figure 8 shows the influence of electrolyte conductivity on the average velocity u_ave_ obtained with and without slip velocity for *L*_s_ = 0.5 and 1 µm. In all the studied cases, the average velocity u_ave_ exhibited practically a linear increase against the electrical conductivity. For a fixed value of the conductivity, the discrepancies between velocity profile with and without slip velocity become more and more important when *L*_s_ increases. More precisely, for the same value of the conductivity, the discrepancy on the average velocities obtained with and without slip velocity is about unity when *L*_s_ = 0.5 µm and increases to 2 for *L*_s_ = 1 µm. These curves prove that the average velocity depends on many parameters. Until now, the effects of the presence or absence of slip velocity, the applied voltage, and the electrolyte conductivity have been revealed. The effects of other parameters will be discussed hereafter.

### 4.4. Effects of Slip Velocity and Thermal Conductivity on Average Pumping Velocity

**Figure 9:**
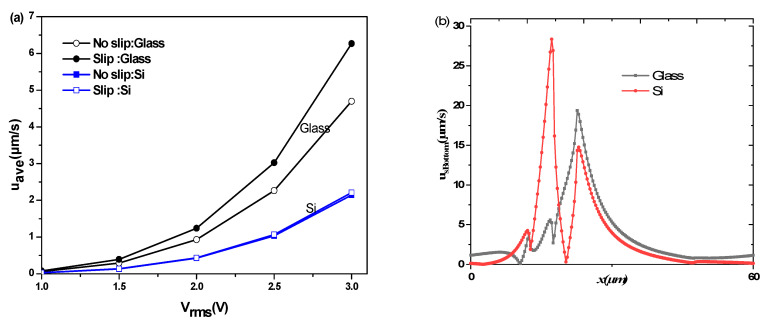
(**a**) Variation of the average velocity versus the rsm voltage for two kinds of substrates. (**b**) Axial profile of the bottom wall velocity for *L*_s_ = 1 µm and for two kinds of substrates.

To investigate the effect of the thermal conductivity on the average velocity, two substrates were used. One was made of glass, whereas the second was made of silica. Figure 9a exhibits the average velocity versus rms voltage for the two substrates when the slip condition is or is not taken into account. For the high thermal conductivity of the substrate (case of silicon λ_Si_ = 150 W/K·m), more heat will be dissipated through this substrate, causing a lower temperature gradient. On the other hand, when λ is low (case of silicon λ_Glass_ = 1.38 W/K·m), the heat flux will be accumulated in the microchannel leading to an increase of temperature gradient.

Figure 9b depicts the axial profiles of the bottom velocities for the two substrates. When the glass is used as a substrate, we notice:

- A significant increase in average velocity.
- The effect of the slip velocity is very significant with a rate of increase of 33%. However, this change is only 3% for silicon.
- A significant increase of the slip velocity at the bottom wall of the microchannel.

### 4.5. Effects of Slip Velocity and Frequency on Average Pumping Velocity

**Figure 10:**
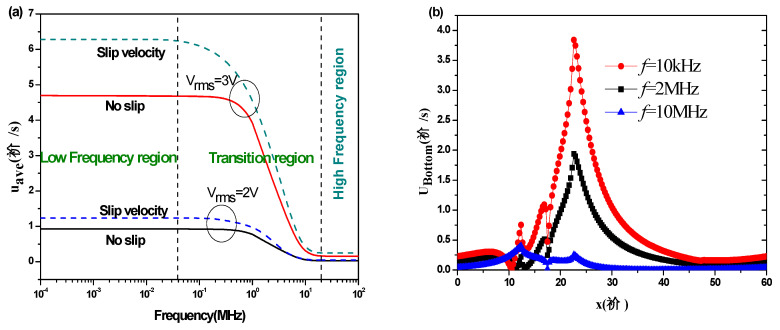
(**a**) Average pumping velocity versus the AC frequency and (**b**) slip velocity along the bottom wall of microchannel for several AC frequencies.

Figure 10a shows the average pumping velocity u_ave_ at different AC frequencies for *V*_rms_ = 2 V and 3 V, with and without slip velocity for the case of *L*_s_ = 1 µm, and σ = 0.01 S/m. In this part, glass was used as a substrate. The diagrams in Figure 10a can be divided into three regions. The first region is related to low frequencies. The second one is related to intermediate frequencies and called the transition region. The third part is related to high frequencies. It appears noticeably that u_ave_ (*f*) takes high values in the low-frequency region while in the high-frequency range it takes much lower values. On the other hand, u_ave_ (*f*) takes a constant value independent of the frequency, whether in the low-frequency range or the high-frequency range. It varies clearly on the frequency only in the transition zone. For low-frequency region I, the Coulomb force is dominant, while for high-frequency region III, where the velocity becomes almost zero, the dielectric force is dominant.

The Coulomb force F_C_, the first term in Equation (5), is due to the net charge induced by the electrical conductivity gradient in an electrical field which is related to the AC frequency. For low frequency, the free electric charge is almost saturated, and as a result the dominant Coulomb force is almost constant and the electrothermal flow velocity is relatively large. On the other hand, when the frequency increases significantly, the free electric charge density increases, and subsequently the effect of the Coulomb force decreases. In this high frequency domain, the electrothermal flow velocity is relatively small.

The effects of slip velocity and applied voltage are much larger at low frequencies. For the low-frequency region I and *V*_rms_ = 2 V, the average pumping velocity is approximately 1.24 µm/s when a slip velocity condition is used, which is 33% greater compared to the flow without slip velocity, which is approximately 0.93 µm/s. In the same low-frequency region, for *V*_rms_ = 3 V, u_ave_ is approximately 6.28 µm/s when a slip velocity condition is used, which is 34% greater compared to the flow without slip velocity, which is approximately 4.70 µm/s. The rate of increase depends obviously on the slip length L_s_.

The effect of AC frequency on the slip velocity along the bottom wall is depicted in Figure 10b. There is a significant reduction of slip velocity when the AC frequency increases. For the low frequency (*f* = 10 kHz), the peak of slip velocity at the bottom wall is great where the shear stress is large. For high frequencies, we notice a significant decrease in the slip velocity.

## 5. Conclusions

This work presented a physical model to describe the AC electrothermal flow in a microchannel. The existence or not of a slip velocity at the microchannel walls was considered. The governing equations have been solved numerically with the finite element method. In addition to the presence or not of slip velocity, the effects of the electrical conductivity of the liquid, substrate material, voltage, and frequency have been investigated. The main findings concerned are listed hereafter.

- Shear stress increases with increasing slip length and applied voltage.
- Slip velocity in the region between the two electrodes is very higher than the slip velocity at the top wall.
- The effect of the slip velocity is very significant with a rate of increase of 33% of the average pumping velocity when a glass substrate is used. However, for a silicon substrate, this rate is only 3%.
- The electrothermal flow does not depend on frequency but depends on the slip velocity and the applied voltage for the frequency range below 100 kHz.

This investigation may be helpful for further studies on ACET micropumps. It is interesting to note that the slip velocity condition has a significant influence on pumping efficiency.

In future work, the findings obtained by FEM simulations shall be confirmed by experimental data. Micropump design considerations, based on these findings, could also be more explicitly provided.

## Figures and Tables

**Table 1:** Physical properties of the fluid, cover, and substrate.

Property	Fluid (KCl)	Cover (PDMS)	Substrate	
			Glass	Silica
Thermal conductivity λ (W/K·m)	0.61	0.18	1.38	150
Relative permittivity ε_r_	80	-	-	-
Electrical conductivity σ (S/m)	0.001–0.1	-	-	-
Dynamic viscosity μ (Pa·s)	10^−3^	-	-	-
